# The Rationale for Insulin Therapy in Alzheimer’s Disease

**DOI:** 10.3390/molecules21060689

**Published:** 2016-05-26

**Authors:** Samo Ribarič

**Affiliations:** Institute of Pathophysiology, Faculty of Medicine, University of Ljubljana, Zaloška 4, SI-1000 Ljubljana, Slovenia; samo.ribaric@mf.uni-lj.si; Tel.: +386-1-543-7053; Fax: +386-1-543-7021

**Keywords:** Alzheimer’s disease, diabetes, insulin, cognition

## Abstract

Alzheimer’s disease (AD) is the most common form of dementia, with a prevalence that increases with age. By 2050, the worldwide number of patients with AD is projected to reach more than 140 million. The prominent signs of AD are progressive memory loss, accompanied by a gradual decline in cognitive function and premature death. AD is the clinical manifestation of altered proteostasis. The initiating step of altered proteostasis in most AD patients is not known. The progression of AD is accelerated by several chronic disorders, among which the contribution of diabetes to AD is well understood at the cell biology level. The pathological mechanisms of AD and diabetes interact and tend to reinforce each other, thus accelerating cognitive impairment. At present, only symptomatic interventions are available for treating AD. To optimise symptomatic treatment, a personalised therapy approach has been suggested. Intranasal insulin administration seems to open the possibility for a safe, and at least in the short term, effective symptomatic intervention that delays loss of cognition in AD patients. This review summarizes the interactions of AD and diabetes from the cell biology to the patient level and the clinical results of intranasal insulin treatment of cognitive decline in AD.

## 1. Amyloidogenesis in Alzheimer’s Disease and Diabetes

The increase in life expectancy in the developed world is accompanied with an increased number of patients suffering from two age-related diseases, Alzheimer’s disease (AD) and diabetes mellitus. AD is the most common form of dementia, with a prevalence that increases with age from 3% in people aged 65–74 to about 50% in people aged 85 or older. The worldwide number of patients with AD is projected to increase from 48 million in 2015 to more than 140 million in 2050 [1]. AD is associated with progressive memory loss, a gradual decline in cognitive function and a premature death 3–9 years after diagnosis [2]. Typical pathological features in the brain of AD patients are (a) intra-neuronal neurofibrillary tangles (NFT) of predominantly hyper phosphorylated tau protein; (b) extracellular deposition of senile plaques mainly composed of aggregated amyloid β (Aβ) peptide and (c) neuronal atrophy, starting in the entorhinal region and the temporal lobe, and progressing to the limbic system and major areas of the neocortex, as reviewed in [3,4,5,6,7]. Most patients with AD have a sporadic, late onset form, where the major risk factors are aging, type 2 diabetes (T2D) and apolipoprotein E ε4 (APOE-ε4) [8,9,10,11,12,13]. The minority of AD patients have the early onset, genetic, familial form of AD due to the presence of autosomal dominant mutations in three genes: amyloid β precursor protein (AβPP), presenilin-1 (PS1), and presenilin-2 (PS2) [14]. Therefore, the cause of disease in most AD patients is not known, what is known are the factors that may increase (e.g., aging, T2D) or decrease (e.g., mental and physical exercise) the risk and progression of AD.

Diabetes mellitus is one of the most prevalent metabolic disorders, with the total number of affected people projected to rise to 366 million in 2030 [15]. Diabetes mellitus is characterized by chronic hyperglycaemia and associated with long-term damage, dysfunction, and failure of various organs, including the brain [16]. Diabetes has been associated with brain atrophy, white matter abnormalities, cognitive impairment, and is also a risk factor for dementia [17,18,19,20,21]. About 90% of diabetes patients have type T2D. Several studies concluded that T2D patients have an increased risk to develop dementia and AD [13,22,23]. It has been suggested that diabetes accelerates the progression of AD, rather than increasing the risk for AD [24]. This view was supported by *post-mortem* studies concluding that, compared to control subjects, senile plaques were less frequent and cerebrovascular pathology was more frequent in T2D patients [23,25]; in animal studies of diet-induced obesity with T2D cognitive impairment, brain atrophy, brain insulin resistance, neuro-inflammation, oxidative stress, and deficits in cholinergic function were relatively mild compared to the expected AD related pathology [26,27,28].

AD and T2D share the process of amyloidogenesis where a soluble protein forms insoluble fibrillary protein aggregates [29]. In AD, extracellular senile plaques in the brain are formed by abnormal protein processing of peptides of 30–51 amino acid residues by the proteolytic cleavage of amyloid β precursor protein (AβPP) by β- and γ-secretases [30]. The most common soluble amyloid β peptides are Aβ42 and Aβ40; the former is produced by cleavage in the endoplasmic reticulum, the latter by cleavage in the trans-Golgi network [31,32,33]. It has been suggested that soluble amyloid β peptides present a negative feedback loop regulating synaptic plasticity and neuronal survival since low concentrations of Aβ are present in the central nervous system of non-demented individuals [34]. Also, in cell culture studies, low concentrations of Aβ were neurotrophic to undifferentiated hippocampal neurons and neurotoxic to mature neurons at higher concentrations [35]. Under pathological conditions, Aβ42 and Aβ40 form toxic soluble oligomers (AβOs) that lead to cell death; Aβ42 is more susceptible to conformational changes than Aβ40 [36]. Studies of patients with AD, and of AD animal models, have linked AβO with synaptic dysfunction, cognitive decline, inhibition of hippocampal long-term potentiation (LTP) component in memory, and learning and memory impairment [37,38,39,40,41,42,43,44,45,46]. In these studies, AβOs were better correlated with dementia and synaptic loss then Aβ in extracellular amyloid plaques [37,38]; however, toxicity mechanisms of amyloid and AβOs were reported to be similar [47,48,49]. Several factors contribute to the progression of AD. What is not clear is the sequence of events that initiate the transition from a disease free state to an irreversible, progression of AD. Therefore, one of the key questions “*In a normal human brain,* w*hat causes the increase in soluble amyloid* β *peptides and their transition to AβOs?*” has yet to be answered.

In human pancreatic islets of T2D patients, there is an accumulation of fibrillary protein aggregates of amylin—human islet amyloid polypeptide (hIAPP), a 37 residue peptide hormone, that is secreted from pancreatic β-cells in conjunction with insulin [50,51,52]. Human amylin forms oligomers that bind to AβOs antibodies suggesting a common conformation [53,54,55]. The molecular structure and morphology of amylin fibrils resemble Aβ fibrils of AD [56]. Amylin oligomers, the building blocks of amylin fibrils, induced β-cell apoptosis [57,58,59] and their toxic effect on cultured β-cells was similar to the AβOs’ toxic effect in neurons [48,59,60,61,62]. Amylin accumulation in the pancreas is associated with a reduced β-cell volume and is present in the pancreatic islets of 90 % T2D patients [56,63]. Although several contributing factors have been suggested, the mechanism of transition from soluble amylin to toxic amylin aggregates is not known [64,65,66,67,68,69,70,71]. A step towards understanding the development of fibrillary protein aggregates of hIAPP was made by the study in isolated islets from hIAPP transgenic mice where amylin accumulated in a time- and glucose-concentration-dependent fashion and was associated with decreased β-cell areas and increased β-cell apoptosis [72,73]. Amylin oligomeric and plaque-like accumulations in brain parenchyma and cerebral vasculature were detected in T2D patients and in nondiabetic patients with late onset AD where amylin plaques were usually not co-localized with Aβ plaques. The authors suggested that amylin amyloid formation in the wall of cerebral blood vessels could contribute to a reduced elimination of Aβ from the brain, thus contributing to the progression of AD [74]. In an animal rat model, overexpressing human amylin in the pancreas, the elicited hyperamylinemia promoted accumulation of oligomerized amylin in the rat’s brain, associated with an amylin-mediated brain inflammatory response, a reduced exploratory drive and a poor vestibulomotor performance on the rotarod test [75].

To summarize, T2D and AD are amyloid-forming diseases with insoluble protein aggregates in a fibrillary conformation that are caused by amylin deposition in pancreas and Aβ deposition in brain, respectively. Amylin aggregation is associated with pancreatic β-cells loss, while Aβ aggregation is associated with neuronal and synaptic dysfunction.

## 2. Interactions between Pathological Mechanisms of Alzheimer’s Disease and Diabetes

The pathological mechanisms of AD and diabetes interact and tend to reinforce each other at the level of reduced cerebral blood flow and altered glucose metabolism, impaired insulin signalling, mitochondrial dysfunction, oxidative stress, advanced glycation end products, altered cholesterol metabolism, inflammation and cognitive impairment [3,5,6,7,16,76].

### 2.1. Cerebral Blood Flow and Glucose Metabolism

A decreased cerebral blood flow and a reduced brain glucose uptake occur during normal ageing of the human brain. These changes are more pronounced and occur sooner in life in patients with AD, in T1D and T2D patients. A reduced cerebral blood flow, by itself, promotes Aβ accumulation in the brain and increased Aβ brain levels promote local vasoconstriction, thus further promoting Aβ accumulation.

#### 2.1.1. Cerebral Blood Flow and Glucose Metabolism in Alzheimer’s Disease

**Human studies.** The normal ageing process of the brain is characterised by a progressive increase of morphologically abnormal capillaries, a decreased cerebral blood flow (CBF) and a lower brain glucose uptake and metabolism [77,78,79,80]. These changes develop sooner and to a greater degree in patients with AD [78,79,80,81,82,83]. PET studies associated impaired brain glucose metabolism with AD pathology, suggesting a causal link between impaired brain glucose metabolism and cognitive symptoms in AD patients [84]. A reduced brain glucose metabolism in AD patients in the early stages is most prominent in the posterior cingulate and parieto-temporal regions, and spreads to the prefrontal cortex in the advanced stages of the disease [85]. The CBF of AD patients was progressively reduced by 20% in the early stages of the disease and later by 55%–65% in the advanced stages of the disease [86]. Of interest is the finding that young and middle aged APOE-ε4 allele carriers have normal cognition but also abnormally low rates of glucose metabolism, in the same brain regions as patients with probable AD, decades before the possible onset of dementia [87]. The glucose transporter GLUT-1 is significantly reduced in aged humans and in AD transgenic mice, coinciding with hippocampal atrophy [88]. In brains of AD patients, there was a negative correlation between on the one hand decreased GLUT-1 and -3 levels, decreased hypoxia-inducible factor 1-alpha (HIF-1α) and decreased O-GlcNAcylation and on the other hand hyper phosphorylation of tau protein and increased density of NFTs; concomitantly, the level of GLUT-2 was increased due to astrocyte activation [89].

**Animal model studies.** surgical reduction of CBF in an AD knock-in mouse model precipitated a positive-feedback cycle between brain Aβ accumulation, cerebral amyloid angiopathy, amyloid plaque deposition and cognitive impairment on the one hand and CBF reduction on the other [90]. In a mouse model, the binding of Aβ with the receptor for advanced glycation end products (RAGE) on the blood-brain barrier (BBB) triggered the release of vasoconstrictor endothelin-1 and pro-inflammatory factors thus reducing CBF [91].

#### 2.1.2. Cerebral Blood Flow and Glucose Metabolism in Diabetes

**Human studies.** A reduced cerebral glucose metabolism and insulin resistance were associated with memory deficits in pre-diabetic and T2D patients [92]. In human, insulin resistance was associated with a reduced cerebral glucose metabolism in frontal, temporo-parietal and cingulate regions in cognitively intact adults with prediabetes or T2D [92]. T1D patients, treated with insulin for several years, had increased cerebrospinal fluid (CSF) concentrations of soluble low density lipoprotein receptor-related protein (LRP1) [93] that promoted the removal of Aβ from the brain. T1D patients with recurrent hypoglycaemia and chronic hyperglycaemia were at an increased risk of cognitive decline [3].

**Animal model studies.** The expression of LRP1, a BBB transporter of Aβ from the CSF into the blood, was down regulated in brain capillaries of streptozotocin-injected mice [94]. In streptozotocin injected mice, insulin reduced the concentration of RAGE in isolated brain micro vessels [95]. An animal model of diabetic AD mice, for investigating the links between T2D and AD, could be created with the combination of feeding a high-fat diet (HFD) to mice overexpressing AβPP [5].

### 2.2. Impaired Insulin Signalling Links Systemic and Brain Oxidative Stress, Inflammation, Impaired Memory and Insulin Resistance in Diabetes and Alzheimer’s Disease

Insulin modulates neurotransmitter release and synaptic plasticity, the basis for cognition, learning and memory [96,97,98,99,100,101]. Animal models and studies on patients have extensively documented impaired insulin signalling and degradation in AD and diabetes. Animal and cell culture models correlated reduced insulin signalling with increased activation of glycogen synthase kinase 3 beta (GSK3β), hyper phosphorylation of tau protein, increased levels of Aβ and cognitive deficiencies. In animal models, the dysregulated GSK3 activity contributed to both diabetes [102,103] and to AD [104]. Soluble Aβ (40) is a competitive inhibitor of insulin binding to the insulin receptor (IR), and increased levels of this Aβ could contribute to impaired insulin signalling and cognitive impairment in patients with AD. Both increased or decreased insulin blood levels can have detrimental effects on the progression of AD. In T2D, increased insulin blood levels promoted Aβ accumulation by insulin competing with Aβ for insulin degrading enzyme (IDE) [6,105]. IDE degrades both insulin [106] and Aβ *in vivo* and *in vitro* [107,108]. Insulin deficiency, (in T1D or in the latter stages of T2D) attenuates insulin’s and insulin-like growth factor-1’s (IGF-1’s) inhibition of AβOs’ binding to insulin receptor, thus reducing their protection of synapses from AβOs’ toxic effects [109,110].

**Figure 1:**
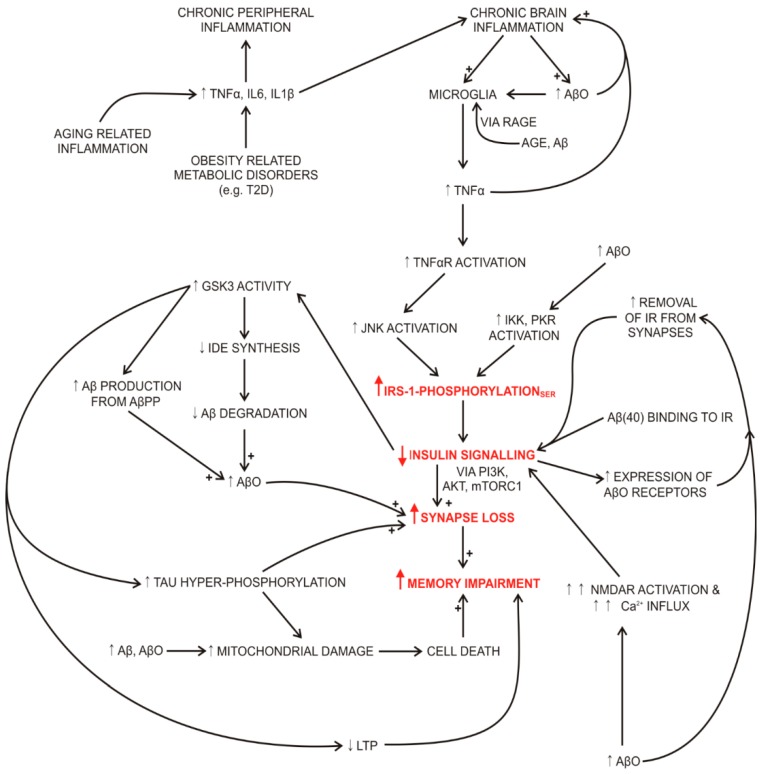
Interactions between AD and T2D signalling pathways in the brain. Abbreviations: Aβ (amyloid β peptide); AβO (toxic, soluble oligomer); AβPP (amyloid β precursor protein); AGE (advanced glycation end product); AKT (protein kinase B); GSK3 (glycogen synthase kinase 3); IDE (insulin degrading enzyme); IKK (IκBα kinase); IL1β (interleukin 1β); IL6 (interleukin 6); IR (insulin receptor); IRS-1 (insulin receptor substrate 1); JNK (c-Jun N-terminal kinase); LTP (long-term potentiation); mTORC1 (mammalian target of rapamycin complex 1); NMDAR (*N*-methyl-d-aspartate receptor); PI3K (phosphoinositide 3-kinase); PKR (double-stranded RNA-dependent protein kinase); RAGE (receptor for advanced glycation end products); SER (serine residue of IRS-1); T2D (type 2 diabetes); TNFα (tumour necrosis factor α); TNFαR (tumour necrosis factor α receptor).

Chronic, low-intensity systemic inflammation, for example during the ageing process or in T2D is characterised by increased blood levels of tumour necrosis factor α (TNFα) and interleukins 1β and 6. These peripherally released inflammatory mediators cross the blood–brain barrier and contribute to brain inflammation. Brain inflammation is further exacerbated by advanced glycation end products (AGE) and amyloid β-peptides (Aβs) in the brain that bind to RAGE and elicit the release of TNFα, IL1β and IL6 from microglia thus further increasing the brain levels of these inflammatory mediators. TNFα binds to its receptor on neurons and activates c-Jun N-terminal kinase (JNK). This kinase phosphorylates insulin receptor substrate 1 (IRS-1) at serine residues, which leads to the dissociation of IR from IRS1 and prevents further tyrosine phosphorylation of IRS-1 by the insulin-activated IR. The attenuated insulin signalling leads to reduced phosphoinositide 3-kinase (PI3K), protein kinase B (AKT) and mammalian target of rapamycin complex 1 (mTORC1) activities, which inhibit the development of synaptic plasticity and memory formation directly and also indirectly by an increased activity of glycogen synthase kinase 3 (GSK3). Brain insulin resistance in AD, due to chronic, low-intensity systemic inflammation, if further compounded by the presence of increased levels of Aβs and AβOs in the brain. Aβs bind not only to RAGE on microglia, further promoting brain inflammation, but also to the IR, diminishing insulin signalling by preventing insulin binding. AβOs’ accumulation in the brain was associated with (a) increased brain levels of TNFα; (b) removal of IRs from the synapses and their redistribution to the neuron’s cell body; (c) decoupling of IRS-1 from IR in neurons that is mediated by IκBα kinase (IKK) and double-stranded RNA-dependent protein kinase (PKR) signalling and (d) aberrant binding to the *N*-methyl-d-aspartate receptors (NMDARs) that stimulated excessive Ca^2+^ influx, increased oxidative stress and possibly activated protein tyrosine phosphatases that could further inhibit IRS-1 signalling [111,112,113,114,115,116]. The interactions between AD and T2D signalling pathways in the brain are summarized in Figure 1.

#### 2.2.1. Impaired Insulin Signalling and Degradation in Alzheimer’s Disease

**Human studies.** In AD patients, the insulin dose response curve for memory had an inverse U shaped function, with beneficial effects observed at the apex and null or negative effects when levels were too low or too high [117]. AD patients had reduced brain insulin receptor (IR) activity, lower CSF insulin levels and peripheral blood hyperinsulinemia [85,118] and an attenuated expression of IR and IGF receptors [119]. mRNA levels of insulin, IGF and their receptors were reduced in *post-mortem* human AD brains compared with controls [119] and this reduction was progressive with increasing severities of AD Braak Stage [120]. Insulin treatment, without changing fasting plasma glucose level, enhanced memory performance in AD patients [121,122].

**Animal model studies.** In a transgenic mouse model of AD, attenuated IR signalling reduced signalling through the phosphoinositide 3-kinase-protein kinase B (PI3K-Akt) pathway, increased activation of GSK3β and hyper phosphorylation of tau protein [123,124,125]. Overexpression of GSK3β in the brain of transgenic mice was associated with an increased level of hyper phosphorylated tau and cognitive deficiencies [126]. Inhibition of GSK3β reduced Aβ and hyperphosphorylated tau-associated neurodegeneration both *in vivo* and *in vitro* [127,128,129,130]. Cell culture models demonstrated that soluble Aβ (40) is a competitive inhibitor of insulin binding to the IR [131,132].

#### 2.2.2. Impaired Insulin Signalling and Degradation in Diabetes

**Animal model studies.** T1D animal model studies demonstrated: (1) an association between impaired cognitive performance and reduced hippocampal plasticity [133]; (2) a progressive impairment of cognitive function with an impairment of insulin and IGF-1 actions and neuronal apoptosis in hippocampus [134]; (3) mitigation of cognitive dysfunction and hippocampal apoptosis by proinsulin C-peptide with no concomitant effect on glucose levels [135]; (4) a loss of pancreatic β cells and long-term cognitive behaviour deficits in intra-cerebro-ventricularly streptozotocin treated rats [125,133,136]; (5) insulin treatment prevented streptozotocin induced deficits in the rat’s cognition [99]; (6) the major contributing factor of T1D to AD was insulin deficiency that promoted increased tau protein phosphorylation in the cortex and hippocampus [137]. T2D animal model studies demonstrated that: (1) neuronal loss and neurite degeneration, associated with altered AβPP metabolism, hyper phosphorylation of tau protein, and impaired signalling of insulin and IGF-1, were more severe in rat models of T2D than in rat models of T1D [138]; and (2) in a T2D animal model, the major contributing factor to AD was hyperglycaemia-mediated proteolytic tau cleavage since the cleaved tau served as a nucleation centre for the pathological assembly of tau filaments [137].

### 2.3. Mitochondrial Dysfunction in Alzheimer’s Disease and Diabetes

Mitochondrial (MITO) dysfunction has a key role in the pathogenesis of AD, T1D and T2D [139,140]. Mitochondrial dysfunction precedes and sustains Aβ accumulation in AD patients. In isolated human, rat and mouse MITO models, Aβ (40 or 42) inhibited MITO enzymes cytochrome c oxidase and α-ketoglutarate dehydrogenase, leading to MITO impairment [141,142,143]. Also Aβ and AβOs accumulated in mitochondria of transgenic mice overexpressing mutant AβPP and in *post-mortem* brains from AD patients [142,143,144,145]. Studies on transgenic mice and AD patients confirmed that Aβ can directly interact with MITO Aβ-binding alcohol dehydrogenase (ABAD) leading to increased ROS generation, MITO dysfunction and cell death [146]; inhibition of the ABAD–Aβ interaction in a mouse model attenuated Aβ accumulation, conserved MITO function and improved spatial learning in an AD animal model [147]. In summary, the pathological basis of MITO dysfunction in AD, T1D and T2D is a combination of oxidative modifications of key MITO enzymes (e.g., pyruvate dehydrogenase, isocitrate dehydrogenase, α-ketoglutarate dehydrogenase and cytochrome c oxidase), reduced antioxidant defences and an increased production of ROS.

#### 2.3.1. Mitochondrial Dysfunction in Alzheimer’s Disease

**Human studies.** The key findings on MITO dysfunction in AD were: (1) MITO dysfunction was observed in platelets and *post-mortem* brains of AD patients [148,149,150,151,152,153,154]; (2) MITO enzymes pyruvate dehydrogenase, isocitrate dehydrogenase, and α-ketoglutarate dehydrogenase isolated from fibroblasts and brain tissue of AD patients were more susceptible to oxidative modification [155,156] and were altered by exposure to several pro-oxidants [157] and (3) in normal astrocytes, from primary human cortical foetal cell cultures, the inhibition of MITO metabolism by a MITO uncoupler induced amyloidogenic AβPP processing and Aβ accumulation seen in Down’s syndrome of the brain [158].

**Animal model and cell culture studies.** The key findings on MITO dysfunction in AD were: (1) cells depleted of endogenous and repopulated with platelet MITO DNA from AD patients expressed MITO dysfunction, *i.e.*, reduced cytochrome c oxidase activity and enhanced ROS production [159]; (2) MITO dysfunction in cortices of transgenic AD mice preceded formation of amyloid plaque and NFT [160]; (3) in a transgenic AD mouse model, knockout of manganese superoxide dismutase, a major MITO antioxidant enzyme, increased Aβ levels and amyloid plaque formation in the brain [161]; and (4) full-length AβPP accumulated in MITO of cortical neurons and this accumulation precipitated MITO dysfunction [162].

#### 2.3.2. Mitochondrial Dysfunction in Diabetes

**Human studies, animal model and cell culture studies**. Patients with T2D have altered MITO morphology and deficiency in bioenergetics and antioxidant capacity [163,164]. MITO dysfunction was demonstrated in animal models of diabetes [165,166,167,168,169]. In a T1D animal model, acute insulin-induced hypoglycaemia potentiated the detrimental effects of chronic hyperglycaemia in cortical and hippocampal MITO: the increase in ROS levels and decrease antioxidant defences [140]. Also, nerve damage, observed in an animal model of T1D, was causally linked to an increased, Ca^2+^-independent release of the excitatory amino acid glutamate during acute insulin-induced hypoglycaemia or during chronic hyperglycaemia [170].

### 2.4. Oxidative Stress

Increased oxidative stress is present in AD, T1D and T2D. In AD, oxidative stress precedes and coincides with Aβ plaque formation, suggesting its role in initiating and sustaining AD-related changes in the brain. Oxidative stress promotes Aβ production by upregulating β-secretase and γ-secretase expression [171,172,173,174,175,176]. Aβ interacts with MITO proteins, disrupting the electron transport chain and promoting MITO dysfunction and an increased generation of ROS [177]. Oxidative stress also enhances tau hyper phosphorylation and subsequent NFT formation [178].

#### 2.4.1. Oxidative Stress in Alzheimer’s Disease

**Human studies and animal models.** Oxidative damage is increased in brain tissue samples from patients with AD [179,180,181,182,183,184,185,186]. Human autopsy brain samples from patients with AD and animal models of AD imply that oxidative damage occurrs before Aβ plaque formation [187,188,189]. In a transgenic AD mouse model, an increase in reactive nitrogen species coincided with the onset of Aβ deposition [190].

#### 2.4.2. Oxidative Stress in Diabetes

**Human studies, animal models and cell culture studies.** Clinical trials, as well as animal and cell culture models of diabetes, demonstrate that hyperglycaemia promotes an excessive and generalised production of free radicals in T1D and T2D [191,192,193].

### 2.5. Advanced Glycation End Products (AGEs)

The production of AGEs is enhanced by chronic hyperglycaemia in diabetes and by chronic oxidative stress present in diabetes and AD. Aβ and AGEs bind to RAGE on microglial cells thus stimulating the release of proinflammatory mediators, (*i.e.*, free radicals and cytokines) and promoting the development of amyloid plaques and NFT [194,195,196]. AGEs and oxidative stress independently cause pathological changes on macromolecules and also act synergistically thus potentiating protein damage [197,198].

#### 2.5.1. Advanced Glycation End Products in Alzheimer’s Disease

**Human studies and cell culture studies.** In brain tissue from patients with AD, AGEs co-localized with NFT and amyloid plaques [199]. Also, AD plaques have more AGEs than healthy, age-matched controls [200]. It has been suggested that AGEs’ accelerated the aggregation of both soluble Aβ and tau thus facilitating development of NFTs and amyloid plaques [200,201]. In cultured neuroblastoma cells, AGEs promote neuronal oxidative stress and inflammation by nuclear factor kappa-light-chain-enhancer of activated B cells (NF-κB) activation, increased cytokine IL6 gene expression and increased Aβ release [202].

#### 2.5.2. Advanced Glycation End Products in Diabetes

**Human studies.** In T2D patients, the formation and accumulation of AGEs, present in normal aging, is accelerated thus leading to diabetes associated damage on the retinal, renal, cardiovascular and peripheral nervous tissue [203,204,205,206,207]. Vascular AGEs staining is associated with cognitive impairment and a history of diabetes [208,209].

### 2.6. Cholesterol Metabolism

Increased blood cholesterol levels stimulate β- and γ-secretase proteolytic activity thus promoting Aβ production and amyloid accumulation in the brain [210,211,212]. Increased blood cholesterol was also identified as an independent risk factor for AD [213]. In the brain, cholesterol is transported to the neurons by apolipoprotein E-ε (APOE-ε), locally synthesized by astrocytes. The normal function of APOE-ε is essential not only for cholesterol catabolism but also for preventing AD-related brain changes. The isoform encoded by the ApoE-ε4 allele promotes tau phosphorylation and inflammation thus contributing to the development of AD [214,215,216,217].

#### 2.6.1. Cholesterol Metabolism in Alzheimer’s Disease

**Human studies.** Caucasian heterozygous and homozygous ApoE-ε4 allele carriers have a three to eight-fold increased risk of AD compared to non ApoE-ε4 carriers [8,218,219]. Also, MITO dysfunction in AD patients with ApoE-ε4 allele correlate better with cognitive dysfunction, than in AD patients carrying the ApoE-ε3 allele [220].

**Animal model studies.** The conclusions of cholesterol metabolism studies are: (1) the reduction of cholesteryl-ester levels, by inhibiting Acyl-CoA cholesterol acyltransferase (ACAT), reduces Aβ production in cultured cells [221]; (2) in cell culture and whole animal mouse models of AD, ACAT1 inhibition reduces amyloid and tau deposition by enhancing autophagy [222,223,224]; (3) genetic ablation of ACAT in an AD mouse model increases cholesterol and 24(S)-hydroxycholesterol contents in the endoplasmic reticulum of mouse brain cells, attenuated human AβPP harbouring the Swedish mutation and 3-hydroxy-3-methylglutaryl-CoA reductase protein contents and ameliorated Aβ pathology [225] and (4) cholesterol depletion in rat hippocampal neurons decreases generation of Aβ [226]. The implication of ApoE-ε in the development of AD was demonstrated by the following studies: (1) in transgenic mice, C-terminal truncated ApoE-ε4 removed Aβ with low efficiency and also acted synergistically with Aβ to contribute to neuronal and behavioural deficits [227]; (2) carboxyl-terminal-truncated apolipoprotein ε4 caused Alzheimer’s disease-like neurodegeneration and behavioural deficits in transgenic mice [228]; (3) ApoE contributed to the clearance of soluble Aβ from the brain interstitial fluid of transgenic mice; the clearance was considerably lower in mice that expressed ApoE-ε4 than in mice that expressed ApoE-ε2 or ApoE-ε3 [229]; in murine ApoE knockout mice, the expression of human ApoE-ε4, but not of ApoE-ε3, lead to deficits in learning and spatial memory, that increased with age and were seen primarily in females, although the Aβ levels in female brains were comparable to male [230,231].

#### 2.6.2. Cholesterol Metabolism in Diabetes

**Human studies.** T2D patients with the ApoE-ε4 allele are two-fold more likely to develop AD than nondiabetic ApoE-ε4 carriers [232].

### 2.7. Inflammation

Inflammation is present in AD, T1D and T2D. Inflammation sustains insulin resistance, results in compensatory increase of insulin levels in T2D, and contributes to the destruction of pancreatic β cells in T1D [233,234,235,236,237,238,239]. In AD, inflammation promotes Aβ deposition and tau hyper phosphorylation thus contributing to the progression of disease [240,241,242,243,244,245,246,247,248,249,250,251].

#### Inflammation in Alzheimer’s Disease

**Human studies.** *Post-mortem* human AD brains have an increased activation of inflammatory and immune pathways with upregulated levels of pro-inflammatory cytokines, chemokines and complement proteins [240]. This is consistent with microarray studies of brain samples from humans with AD or from animal models of AD that have identified an increased expression of genes involved in inflammation [242,243]. The intensity of inflammation varies over the course of AD. In patients with AD, cytokines in ventricular fluid were activated early but not late in the clinical course of AD; the observed reduced cytokine activation overlapped with reduced expressions of trophic factor and mediators of insulin signalling/responsiveness, and was concomitant with the increased brain levels of Aβ, tau, and AGEs [241]. Treatment with nonsteroidal anti-inflammatory drugs (NSAIDs) was epidemiologically associated with a reduction in AD risk [246]; however, randomized trials failed to validate the benefit of several anti-inflammatory drugs in patients with AD [248,249,250,251].

**Animal model studies.** AD animal models have increased microglial activation and inflammation in the brain [244]. An AD animal model of AβPP transgenic mice, demonstrated that inflammation promoted Aβ deposition; an increased expression of β-site amyloid precursor protein cleaving enzyme 1 (BACE1) was associated with inflammation and this inflammation preceded Aβ deposition [245]. Chronic administration of lipopolysaccharide lead to increased IL-1 levels and tau hyper phosphorylation in a triply transgenic AD mouse model [246].

### 2.8. Cognitive Impairment and Brain Insulin Sensitivity

The hippocampus is rich with insulin receptors that are localized mainly to nerve synapses [111,252]. It was demonstrated that insulin receptor signalling contributed to long-term memory consolidation and improved spatial learning in an animal model [253,254,255]. These observations are consistent with the suggested insulin involvement in the acquisition, consolidation and retrieval of memories [101]. Cognitive impairment is present in AD, T1D (due to insulin deficiency) and T2D (due to impaired insulin sensitivity of the brain) [256,257,258]. In patients with AD or mild cognitive impairment a single or repetitive administration of intranasal insulin improved memory and cognitive function [259,260,261,262,263]. When compared to the general population, T1D and T2D patients have a more pronounced brain atrophy [264,265,266,267]. The neuroprotective effects of insulin are mediated by (1) insulin binding to IGF receptors 1 and 2 [268] that attenuate GSK3α activity [269] and reduce the neurotoxic effects of AβPP [270,271,272,273]; (2) attenuation of AβOs binding to neurons thus protecting synapses against the toxic effects of AβOs [111] and (3) increasing transcription of antiamyloidogenic proteins insulin-degrading enzyme and α-secretase and decreasing the transcription of pro-amyloidogenic proteins AβPP, β-secretase, and glycogen synthase kinase 3 α (Gsk3α) [274].

**Human studies.** Insulin infusion in normal older adults improves declarative memory and also increases CSF AΒ42 levels thus inhibiting Aβ42 intracellular accumulation by stimulating its extracellular secretion; improvement of declarative memory was attenuated in subjects with the highest, pre-insulin treatment levels of CSF Aβ42 [275]. Impaired insulin sensitivity of the brain was associated with (1) cognitive decline and brain atrophy in healthy elderly men and women [276]; (2) a positive correlation with the extent of cognitive impairment in AD patients [277,278] and (3) an increased risk for AD in women with elevated plasma insulin levels and decreased connectivity between the prefrontal cortex and hippocampus [279]. In *post-mortem* human brain samples of patients with AD the neuronal loss and impaired insulin/IGF signalling mechanisms correlates with the reduced expression of choline acetyltransferase [120].

## 3. Treatment of Alzheimer’s Disease

The results of developing AD therapies on the basis of amyloid and NFTs hypotheses have been disappointing, even when the effective clearing of Aβ deposits in AD brain was demonstrated [128,280,281,282,283,284,285,286]. Alternative therapies with antioxidants [287], anti-inflammatory agents [288,289] were also ineffective. Current therapies of AD are symptomatic using NMDAR antagonists and cholinesterase inhibitors [7]. A personalised therapy approach for AD, based on clinical trials that selectively target different stages of AD, has been suggested as a way forward for promoting effective treatment. For example, at the pre-disease and pre-clinical stages of AD, treatment should be focused on managing factors that contribute to the onset of the disease (e.g., insulin resistance). For treating the pre-dementia stage, different combinations of preventive and curative drugs were suggested to stop the disease progression, and at the dementia stage, drugs targeting multiple pathogenic mechanisms of AD should be employed [7]. Combining different types of drugs, for personalised treatment of patients with AD, opens new challenges since it can lead to side effects and lower efficacy, as in the case of combined therapy with memantine and cholinesterase inhibitors [290].

## 4. Antidiabetic Drugs for Treatment of AD

Insulin contributes to normal brain function, and insulin-signalling dysfunction accelerates the progression of AD as discussed in chapters 1 and 2.2. Therefore, therapeutic agents developed for the treatment of T2DM could be useful for treating AD. Drugs for treating T2D may affect the progression of AD brain changes either indirectly, by modifying the systemic blood concentrations of glucose, insulin, inflammatory markers and AGEs or directly in the brain, provided they pass the blood-brain barrier. Recent drug development for treating AD has focused on diabetes drugs that have a direct effect in the brain tissue since brain insulin resistance is often associated with AD [291].

### 4.1. Peroxisome Proliferator-Activated Receptor-γ Agonists

Thiazolidinediones (TZDs) diabetes drugs increase insulin sensitivity by activating the nuclear receptor peroxisome proliferator-activated receptor-γ, thus increasing the expression of the glucose transporter GLUT-4. Two drugs, rosiglitazone and pioglitazone, are on the market for treatment of T2D; however, their use is restricted by their adverse side effects that include fluid retention, bone fractures and cardiovascular events [291,292]. Although initial studies in humans reported improved biomarkers of AD, improved memory and cognition these findings were not confirmed by larger clinical studies [293,294,295,296]. It was suggested that the potential long term beneficial effects of rosiglitazone, to ameliorate neuronal insulin resistance, were attenuated by its low blood-brain barrier penetration and a pronounced sensitizing effect on peripheral tissues to insulin, with a concomitant decrease in blood insulin levels that leads to a short term decrease in brain insulin signalling and worsening of cognitive impairment [297]. Two pilot studies are in progress and registered at ClinicalTrials.gov to evaluate pioglitazones efficacy for treating mild cognitive impairment due to AD: NCT01931566 with an estimated primary completion date of July 2019; and NCT02284906 with an estimated primary completion date of April 2021.

### 4.2. Metformin

Metformin’s glucose-lowering actions include increased glucose uptake in peripheral tissues and decreased liver gluconeogenesis by activating AMP-activated protein kinase (AMPK) in liver and other tissues. Activation of AMPK could increase insulin sensitivity through interactions with mTOR, P38 mitogen-activated protein kinases (p38 MAPK), and protein kinase C [291]. Animal studies support the suggestion that metformin penetrates the BBB and activates AMPK in the brain [298,299]. In isolated neuronal cells metformin sensitized neurons to insulin and prevented AD pathology in neurons chronically exposed to a hyperinsulinemic environment [300] but also increased β-secretase 1 (BACE1) transcription and generation of amyloid-β [301]. Two pilot studies are registered at ClinicalTrials.gov to evaluate metformin’s efficacy in humans: NCT00620191 that was completed but has not published the results and NCT01965756 with an estimated primary completion date of December 2015.

### 4.3. Glucagon-Like Peptide-1 Receptor Agonists

Glucagon-like peptide-1 receptor agonists (GLP-1) is a hormone structurally unrelated to insulin. It circumvents the upstream stages of the insulin signalling pathway by binding to the G-protein–coupled GLP-1 receptor (GLP-1R) and activating a signalling pathway that converges with the downstream stages of the insulin signalling pathway thus facilitating insulin signalling [291]. GLP-1 stimulates adenylyl cyclase and modulates the activities of protein kinase A (PKA), phosphatidylinositol-4,5-bisphosphate 3-kinase (PI3K), mitogen-activated protein kinase (MAPK), protein kinase C (PKC), and protein kinase B (AKT) [291]. GLP-1 stimulates insulin secretion, decreases glucagon secretion and increases insulin sensitivity without significant hypoglycaemia [292]. GLP-1R is widely expressed in the brain and GLP-1R agonists with prolonged half-lives have been developed and approved for treatment of T2D (e.g., exenatide and liraglutide). The conclusion of preclinical studies of GLP-1R agonists in cell culture and animal models of AD was that GLP-1R agonists promoted synaptogenesis and neurogenesis, protected against oxidative injury, reduced AβO and Aβ plaque load, decreased microglial activation, and improved memory [302,303]. Two ongoing pilot studies are registered at ClinicalTrials.gov to evaluate GLP-1 agonists’ efficacy in humans: NCT01255163 with an estimated study completion date of December 2018 and NCT01843075 with an estimated study completion date of January 2017.

### 4.4. Leptin Analogues

Leptin receptors are abundant in areas of the brain involved in learning and memory and leptin deficiency was linked to cognitive impairment in human studies and in animal models [304,305]. Leptin reduced BACE1 activity, extracellular AΒ levels and tau phosphorylation in AD mouse models [306] and in humans, increased levels of circulating leptin were associated with a reduced incidence of AD [307]. At the time of writing no studies on the efficacy of leptin analogues for treatment of AD are posted on the ClinicalTrials.gov website.

### 4.5. Amylin Analogues

Amylin readily crosses the BBB and the wide distribution of amylin brain receptors implicates amylin in a variety of brain functions, including memory, mood, anxiety, and satiety [291]. Amylin promotes normal blood glucose levels by delayed gastric emptying, decreased glucagon secretion and increased satiety. However, amylin oligomeric and plaque-like accumulations in brain parenchyma and cerebral vasculature were more frequent in patients with diabetes and nondiabetic patients with AD than in normal, nondiabetic control subjects. Amylin plaques were not co-localized with Aβ plaques in most cases [74]. Pramlintide, an amylin analogue, is available as an adjunctive therapy for treatment of T1D and T2D and combines the beneficial glucose lowering effects of amylin without amylin’s propensity to aggregate and form amylin oligomers and plaques and causes only a minimal hypoglycemia. Chronic infusion of pramlintide in an accelerated aging animal model of AD improved memory performance and decreased oxidative stress and inflammatory markers in the hippocampus [308]. Amylin and leptin activate overlapping signalling pathways that converge on the insulin-signalling pathway by activating AKT thus increasing insulin sensitivity. It was suggested that amylin sensitizes neurons to the effects of leptin, because amylin pre-treatment of neurons augmented leptin signalling [309]. Therefore, the synergy of amylin and leptin effects on brain cells could be used to develop combined therapies with amylin and leptin analogues for treatment of AD. At the time of this writing no studies on the efficacy of amylin analogues for treatment of AD are posted on the ClinicalTrials.gov website.

### 4.6. Treatment of Alzheimer’s Disease with Intranasal Insulin Application

The rationale for treating AD with intranasal insulin application was justified by the following research results: (1) insulin receptors were identified in several brain regions, with the highest concentration in the olfactory bulb, hippocampus and hypothalamus [101,310]; (2) with intranasal application, insulin by-passes the BBB and directly enters the brain [311]; (3) insulin was detected in biologically relevant concentrations in the CSF in 30–40 min after intranasal application [312]; (4) intranasal insulin administration was associated with only minor side effects like dizziness or mild rhinitis [313,314,315,316]; (5) AD is associated with brain insulin resistance and insulin deficiency (reduced brain and CSF levels), with or without concomitant systemic insulin resistance or T2DM; (6) patients with diabetes, who were successfully managed with insulin, had a significantly improved memory, an attenuated progression of AD and lower densities of AD-related lesions; and (7) insulin therapy improved memory and cognition in patients with AD [317,318]. The positive effects of insulin therapy diminish with the progression of AD when increased Aβ levels promote brain insulin resistance [319].

Repeated elevations of brain insulin concentrations, by intranasal administration, were associated with reduced Aβ [122], no changes in blood pressure [320], and attenuated hypothalamus-pituitary-adrenal (HPA) secretory activity [259]. Intranasal insulin treatment, for early AD or mild cognitive impairment (MCI), improved memory and attention abilities in four phase 2 clinical trials without significant adverse effects or changes in blood levels of insulin or glucose [117,122,260,263,321,322,323,324,325]. Intranasal insulin administration elicited changes in peripheral glucose metabolism, but no significant change in blood insulin levels were detected [326,327,328,329]. There is some concern, that the efficacy of long-term treatment of AD patients with intranasal insulin application could be attenuated by long-term brain insulin receptor desensitisation or decreased efficacy of peripheral mechanisms (*i.e.*, the “sink hypothesis” for Aβ lowering strategies) that contribute to insulin-induced AΒ clearance from the brain [119,268,277,330,331].

Most of the clinical trials of insulin treatment of AD used the regular, short half-life insulin [122]. An alternative is the insulin analogue detemir with a slower absorption and a higher lipophilicity compared to regular insulin [332,333,334]. Compared to regular insulin, insulin detemir was equal or more effective at reducing hyperglycaemia and nocturnal hypoglycaemic episodes [335] and more effective in eliciting insulin-signalling in the hypothalamus and cerebro-cortical tissue [336] thus generally affecting brain functions to a greater extent than regular insulin [337].

#### Clinical Trials of Alzheimer’s Disease Treatment with Intranasal Insulin Application

All three clinical trials presented below were randomised, blind and placebo-controlled. No treatment-related severe adverse effects were reported in any of these trials.

(a) Single dose trial with regular insulin

The participants were 59 normal adults and 33 memory impaired patients with AD, divided in two subgroups: apolipoprotein E-ε4 alle carriers (ApoE-ε4+) and apolipoprotein E-ε4 alle non-carriers (ApoE-ε4−). Participants received either a placebo or a single dose of 10, 20, 40 or 60 IU of insulin. The changes in plasma insulin, glucose, Aβ42 and Aβ40 levels after insulin treatment were: (1) plasma Aβ40 in normal adults or AD patients was not affected by insulin dosing; (2) plasma insulin and glucose levels in normal adults or AD patients were not influenced by the intervention; (3) plasma Aβ42 levels increased with the increase in insulin dose in ApoE-ε4 − patients; (4) in ApoE-ε4+ patients, there was no significant change in Aβ42 levels with increased insulin concentrations; (5) in normal ApoE-ε4 − adults, the plasma Aβ42 levels showed a U shaped response curve to increasing insulin doses; (6) in normal ApoE-ε4+ adults increasing insulin concentrations had no effect on plasma Aβ.

The effects of insulin treatment on cognition were ApoE-ε4(+/−) dependent: (1) ApoE-ε4− AD patients showed improved verbal memory at the optimal concentration of 20 IU; the insulin-dose response curve for memory had an inverse U shaped function, with beneficial effects observed at the apex and null or negative effects when levels were too low or too high and (2) ApoE-ε4+ AD patients had a decline in verbal memory [117].

(b) Four-months treatment with 20 or 40 IU of regular insulin per day

The participants were 64 adults with amnestic mild cognitive impairment (MCI) and 40 adults with mild to moderate AD. Participants received daily either a placebo, or 20, or 40 IU of insulin for 4 months. The observed changes in Aβ42 cerebrospinal fluid (CSF) levels after insulin treatment were: (1) in both groups of adults, the CSF Aβ42 levels were slightly but not significantly lowered; (2) increased CSF Aβ42 levels were associated with improved delayed memory and preserved caregiver-rated functional ability; (3) decreased tau protein to Aβ42 ratios during the study period were correlated with improved delayed story recall and better daily function; (4) participants receiving 20 or 40 IU dose insulin showed a reduced progression of hypo metabolism in discrete areas of the cerebral cortex compared with the placebo group.

The effect of insulin treatment on cognition was partially insulin-dose dependent: (1) compared to placebo, treatment with 20 IU, but not with 40 IU, improved delayed memory in both groups of patients and (2) treatment with both insulin doses preserved caregiver-rated functional ability and general cognition in both groups of patients [321].

(c) Twenty-one-days treatment with 20 IU or 40 IU of insulin detemir twice daily

The participants were 60 adults with MCI and 20 adults with mild to moderate AD. Both groups had either ApoE-ε4+ or ApoE-ε4− adults. Participants received either a placebo, or 20, or 40 IU of insulin detemir twice daily for 21 days.

The effects of insulin detemir treatment on cognition were: (1) 20 IU insulin detemir treatment did not improve cognitive outcomes in any of the participants; (2) 40 IU insulin detemir treatment did not improve executive functioning or caregiver-rated daily functioning in any of the participants; (3) 40 IU insulin detemir treatment improved: verbal memory for ApoE-ε4+ adults with MCI or AD; visuospatial and verbal working memory for ApoE-ε4+ and APOE-ε4− participants; and peripheral insulin resistance for ApoE-ε4+ adults; and (4) 40 IU insulin detemir treatment increased peripheral insulin resistance in APOE-ε4− participants [316].

## 5. Conclusions

Alzheimer’s disease (AD) is the clinical manifestation of altered proteostasis, the process of accumulating misfolded, pathological proteins in the brain. The cause of altered proteostasis in most AD patients is not known. Known are some of the modulating factors that increase or decrease the risk of AD. The progression of AD is accelerated by several chronic disorders, among which the contribution of diabetes mellitus to AD is well understood at the cell biology level. At present, there is no effective cure for AD, only symptomatic interventions are available. To optimise symptomatic treatment, a personalised therapy approach that selectively targets different stages of AD, has been suggested. Intranasal insulin administration seems to have opened the possibility for a safe and effective symptomatic intervention that delays loss of cognition in AD patients, at least for the short term.
